# Supplementary material for: Deletion of wfs1 Impairs Oligodendrocyte Precursor Cells Dorsal Distribution and Myelination Through the wfs1-hmgcs1 Axis in Zebrafish
Source: Biology (Basel). 2026 Aug 16;15(16):1402. doi: 10.3390/biology15161402 (PMC13510151; doi:10.3390/biology15161402)
Supplement: Supplementary file 1 [file biology-15-01402-s001.zip › File S1-Transcriptome sequencing data and differentially expressed genes (DEGs) in wfs1-- zebrafish at 5 dpf/2.KEGG/wfs1KOvsWT/GSEA/wfs1KO/RNA_DEGRADATION/RNA_DEGRADATION(DRE03018).html]

Details for gene set RNA\_DEGRADATION(DRE03018)[GSEA]

|  || Dataset | deg.sample |
| Phenotype | sample.cls |
| Upregulated in class | wfs1KO |
| GeneSet | RNA\_DEGRADATION(DRE03018) |
| Enrichment Score (ES) | 0.40080723 |
| Normalized Enrichment Score (NES) | 1.9625604 |
| Nominal p-value | 0.0 |
| FDR q-value | 0.028297035 |
| FWER p-Value | 0.01 |
Table: GSEA Results Summary

  

Fig 1: Enrichment plot: RNA\_DEGRADATION(DRE03018)      
 Profile of the Running ES Score & Positions of GeneSet Members on the Rank Ordered List

  

| PROBE | DESCRIPTION (from dataset) | GENE SYMBOL | GENE\_TITLE | RANK IN GENE LIST | RANK METRIC SCORE | RUNNING ES | CORE ENRICHMENT || 1 | 373085\_1 | hspa9 |  |  | 162 | 4.443 | 0.0457 | Yes |
| 2 | 566017\_1 | lsm8 |  |  | 258 | 3.848 | 0.0868 | Yes |
| 3 | 565659\_1 | edc4 |  |  | 304 | 3.620 | 0.1268 | Yes |
| 4 | 492479\_1 | btg3 |  |  | 435 | 3.160 | 0.1589 | Yes |
| 5 | 565000 | exosc3 |  |  | 470 | 3.081 | 0.1931 | Yes |
| 6 | 561416\_1 | pfkpb |  |  | 497 | 2.998 | 0.2265 | Yes |
| 7 | 448949\_1 | cnot1 |  |  | 648 | 2.696 | 0.2527 | Yes |
| 8 | 553665\_1 | cnot6l |  |  | 748 | 2.474 | 0.2779 | Yes |
| 9 | 394064\_1 | exosc10 |  |  | 763 | 2.453 | 0.3055 | Yes |
| 10 | 317641\_1 | zcchc7 |  |  | 875 | 2.275 | 0.3280 | Yes |
| 11 | 100538016 | si:dkey-79d12.5 |  |  | 1149 | 1.957 | 0.3418 | Yes |
| 12 | 321035\_1 | pabpc4 |  |  | 1159 | 1.939 | 0.3638 | Yes |
| 13 | 751632 | exosc5 |  |  | 1460 | 1.686 | 0.3736 | Yes |
| 14 | 30079 | btg2 |  |  | 1650 | 1.568 | 0.3856 | Yes |
| 15 | 100750260\_1 | pnpt1 |  |  | 1722 | 1.524 | 0.4008 | Yes |
| 16 | 560944 | pfkpa |  |  | 4480 | 0.748 | 0.3226 | No |
| 17 | 568001\_3 | pfkmb |  |  | 4822 | 0.702 | 0.3199 | No |
| 18 | 100750260\_2 | pnpt1 |  |  | 5851 | 0.603 | 0.2945 | No |
| 19 | 768119\_1 | cnot7 |  |  | 6880 | 0.502 | 0.2679 | No |
| 20 | 57924 | smx5 |  |  | 7220 | 0.472 | 0.2626 | No |
| 21 | 114432 | btg1 |  |  | 7405 | 0.460 | 0.2621 | No |
| 22 | 449918 | cnot10 |  |  | 7585 | 0.449 | 0.2616 | No |
| 23 | 406245 | tob1b |  |  | 7619 | 0.445 | 0.2656 | No |
| 24 | 504054 | edc3 |  |  | 7846 | 0.436 | 0.2635 | No |
| 25 | 57924\_2 | smx5 |  |  | 8128 | 0.417 | 0.2594 | No |
| 26 | 558487 | eno4 |  |  | 8873 | 0.376 | 0.2403 | No |
| 27 | 360153 | dcp1a |  |  | 9089 | 0.361 | 0.2377 | No |
| 28 | 558857 | exosc1 |  |  | 10166 | 0.292 | 0.2072 | No |
| 29 | 559653 | skiv2l |  |  | 10531 | 0.271 | 0.1988 | No |
| 30 | 386629 | tob1a |  |  | 10808 | 0.254 | 0.1931 | No |
| 31 | 550247 | exosc7 |  |  | 10822 | 0.254 | 0.1956 | No |
| 32 | 100126012\_1 | pan3 |  |  | 11008 | 0.244 | 0.1925 | No |
| 33 | 560362\_1 | dis3l |  |  | 11081 | 0.240 | 0.1930 | No |
| 34 | 100332172 | ttc37 |  |  | 11135 | 0.237 | 0.1941 | No |
| 35 | 791461 | parn |  |  | 11145 | 0.237 | 0.1965 | No |
| 36 | 568678 | papd5 |  |  | 11423 | 0.221 | 0.1903 | No |
| 37 | 394008 | xrn1 |  |  | 11487 | 0.217 | 0.1908 | No |
| 38 | 100009659 | patl1 |  |  | 11532 | 0.214 | 0.1919 | No |
| 39 | 406632 | rqcd1 |  |  | 11894 | 0.196 | 0.1828 | No |
| 40 | 449540 | cnot3a |  |  | 11909 | 0.195 | 0.1845 | No |
| 41 | 569072 | dhx36 |  |  | 12055 | 0.188 | 0.1821 | No |
| 42 | 568176 | dcp1b |  |  | 12129 | 0.183 | 0.1819 | No |
| 43 | 327627 | cnot3b |  |  | 12487 | 0.165 | 0.1726 | No |
| 44 | 282676 | hspd1 |  |  | 13246 | 0.128 | 0.1502 | No |
| 45 | 393827 | wdr61 |  |  | 14073 | 0.089 | 0.1252 | No |
| 46 | novel.808 | - |  |  | 14240 | 0.081 | 0.1209 | No |
| 47 | 393712 | exosc4 |  |  | 14263 | 0.080 | 0.1212 | No |
| 48 | 557829 | papd7 |  |  | 14600 | 0.065 | 0.1113 | No |
| 49 | 792831 | pan2 |  |  | 14896 | 0.051 | 0.1026 | No |
| 50 | 394144 | cnot4b |  |  | 15689 | 0.012 | 0.0779 | No |
| 51 | 321940 | xrn2 |  |  | 15911 | 0.002 | 0.0709 | No |
| 52 | 406795 | skiv2l2 |  |  | 16302 | -0.011 | 0.0588 | No |
| 53 | 334116\_1 | eno1a |  |  | 16552 | -0.019 | 0.0512 | No |
| 54 | 100007655 | LOC100007655 |  |  | 16624 | -0.021 | 0.0492 | No |
| 55 | 560386 | cnot6b |  |  | 16755 | -0.024 | 0.0454 | No |
| 56 | 334116 | eno1a |  |  | 16760 | -0.025 | 0.0455 | No |
| 57 | 327625 | pabpc1l |  |  | 16811 | -0.027 | 0.0443 | No |
| 58 | 606498 | pabpc1a |  |  | 16916 | -0.032 | 0.0413 | No |
| 59 | 100535720 | lsm3 |  |  | 18026 | -0.077 | 0.0073 | No |
| 60 | 100126012 | pan3 |  |  | 18098 | -0.081 | 0.0060 | No |
| 61 | 100007313 | ddx61 |  |  | 18346 | -0.092 | -0.0007 | No |
| 62 | 474329 | c1d |  |  | 18680 | -0.107 | -0.0100 | No |
| 63 | 393121 | dcp2 |  |  | 18713 | -0.109 | -0.0097 | No |
| 64 | 100003553 | dis3 |  |  | 18790 | -0.113 | -0.0108 | No |
| 65 | 450057 | exosc9 |  |  | 18931 | -0.119 | -0.0139 | No |
| 66 | 406788 | cnot8 |  |  | 18995 | -0.121 | -0.0145 | No |
| 67 | 321035 | pabpc4 |  |  | 19111 | -0.127 | -0.0166 | No |
| 68 | 323016 | exosc8 |  |  | 19222 | -0.132 | -0.0186 | No |
| 69 | 550259 | pfklb |  |  | 19460 | -0.142 | -0.0244 | No |
| 70 | 100037372 | cnot2 |  |  | 19473 | -0.143 | -0.0232 | No |
| 71 | 393856 | pabpc1b |  |  | 20092 | -0.170 | -0.0407 | No |
| 72 | 415167 | lsm6 |  |  | 20243 | -0.177 | -0.0433 | No |
| 73 | 563948 | lsm5 |  |  | 21277 | -0.229 | -0.0732 | No |
| 74 | 569055 | cnot4a |  |  | 21508 | -0.241 | -0.0777 | No |
| 75 | 564633 | ddx6 |  |  | 21631 | -0.248 | -0.0787 | No |
| 76 | 57924\_1 | smx5 |  |  | 21899 | -0.262 | -0.0841 | No |
| 77 | 492503 | si:dkey-42i9.4 |  |  | 21923 | -0.263 | -0.0818 | No |
| 78 | 445157 | lsm1 |  |  | 22534 | -0.298 | -0.0976 | No |
| 79 | 449987 | zgc:103759 |  |  | 22590 | -0.301 | -0.0959 | No |
| 80 | 378963 | eno3 |  |  | 23157 | -0.338 | -0.1098 | No |
| 81 | 550234 | exosc2 |  |  | 23270 | -0.346 | -0.1094 | No |
| 82 | 402874 | eno2 |  |  | 23379 | -0.353 | -0.1088 | No |
| 83 | 100750260 | pnpt1 |  |  | 23638 | -0.370 | -0.1127 | No |
| 84 | 57924\_3 | smx5 |  |  | 24690 | -0.429 | -0.1408 | No |
| 85 | 326786\_1 | exosc6 |  |  | 24742 | -0.434 | -0.1374 | No |
| 86 | 492479 | btg3 |  |  | 24828 | -0.440 | -0.1351 | No |
| 87 | 768119 | cnot7 |  |  | 26063 | -0.535 | -0.1678 | No |
| 88 | 570106 | pfkla |  |  | 26195 | -0.547 | -0.1657 | No |
| 89 | 393669 | lsm4 |  |  | 26327 | -0.559 | -0.1634 | No |
| 90 | 560362 | dis3l |  |  | 27067 | -0.636 | -0.1794 | No |
| 91 | 100003553\_1 | dis3 |  |  | 27495 | -0.672 | -0.1851 | No |
| 92 | 324048\_1 | cnot6a |  |  | 27800 | -0.716 | -0.1865 | No |
| 93 | 402850 | dcps |  |  | 27976 | -0.739 | -0.1835 | No |
| 94 | 573047 | lsm7 |  |  | 28206 | -0.773 | -0.1819 | No |
| 95 | 447836 | pfkma |  |  | 28916 | -0.902 | -0.1939 | No |
| 96 | 445224 | mphosph6 |  |  | 29285 | -0.995 | -0.1940 | No |
| 97 | 393668 | eno1b |  |  | 29713 | -1.133 | -0.1945 | No |
| 98 | 317641 | zcchc7 |  |  | 29832 | -1.176 | -0.1848 | No |
| 99 | 373085 | hspa9 |  |  | 30106 | -1.291 | -0.1786 | No |
| 100 | 394064 | exosc10 |  |  | 30406 | -1.451 | -0.1714 | No |
| 101 | 568001\_1 | pfkmb |  |  | 30537 | -1.531 | -0.1580 | No |
| 102 | 565659 | edc4 |  |  | 30927 | -1.888 | -0.1486 | No |
| 103 | 448949 | cnot1 |  |  | 30945 | -1.904 | -0.1273 | No |
| 104 | 553665 | cnot6l |  |  | 31268 | -2.362 | -0.1104 | No |
| 105 | 561416 | pfkpb |  |  | 31341 | -2.509 | -0.0840 | No |
| 106 | 566017 | lsm8 |  |  | 31384 | -2.610 | -0.0555 | No |
| 107 | 324574 | wu:fc33b09 |  |  | 31414 | -2.688 | -0.0256 | No |
| 108 | 568001\_2 | pfkmb |  |  | 31666 | -3.543 | 0.0070 | No |
Table: GSEA details [plain text format]

  

Fig 2: RNA\_DEGRADATION(DRE03018)      
 Blue-Pink O' Gram in the Space of the Analyzed GeneSet

  

Fig 3: RNA\_DEGRADATION(DRE03018): Random ES distribution      
 Gene set null distribution of ES for **RNA\_DEGRADATION(DRE03018)**
